# Evaluation of the safety and efficacy of corifollitropin alfa combined with GnRH agonist triggering in oocyte donation cycles. A prospective longitudinal study

**DOI:** 10.5935/1518-0557.20200033

**Published:** 2020

**Authors:** Ioannis Tsakiridis, Robert Najdecki, Petroula Tatsi, Evi Timotheou, Kallirhoe Kalinderi, Georgios Michos, Andriana Virgiliou, Hakan Yarali, Apostolos Athanasiadis, Evangelos G Papanikolaou

**Affiliations:** 1 3^rd^ Department Ob-Gyn, Aristotle University of Thessaloniki, Thessaloniki, Greece; 2 Assisting Nature, Center of Reproduction & Genetics, Thessaloniki, Greece; 3 Hacetepe University, Ankara, Turkey

**Keywords:** corifollitropin alfa, GnRH agonist triggering, donor, IVF, oocyte donation

## Abstract

**Objective:**

In order to help make the dream of parenthood come true for oocyte acceptors, it is essential that the procedure is not dangerous or unpleasant for oocyte donors. The aim of this study was to identify differences in safety, efficacy and patient acceptability between a traditional stimulation antagonist protocol with recombinant-FSH (rFSH) with hCG-triggering, compared with an innovative antagonist protocol with corifollitropin alfa (Elonva^®^) plus GnRH agonist triggering in oocyte donors.

**Methods:**

A prospective longitudinal study was conducted at an *in vitro* fertilization center in Greece. The same eighty donors underwent two consecutive antagonist stimulation schemes. Primary outcomes were patient satisfaction (scored by a questionnaire) and delivery rate per donor. Secondary outcomes were mean number of cumulus-oocyte-complexes, metaphase II (MII) oocytes and ovarian hyperstimulation syndrome (OHSS) rate.

**Results:**

Donors reported better adherence and less discomfort with the corifollitropin alpha + GnRH agonist-triggering protocol (*p*<0.001). No significant differences were identified in the clinical pregnancy rate per donor (*p*=0.13), the delivery rates, the number of oocytes (*p*=0.35), the number of MII oocytes (*p*=0.50) and the number of transferred embryos, between the two protocols. However, the luteal phase duration was significantly shorter (*p*<0.001) in the corifollitropin alpha + GnRH agonist-triggering protocol. Moreover, three cases of moderate OHSS (3.75%) were identified after hCG triggering, whereas no case of OHSS occurred after GnRH agonist ovulation induction (*p*=0.25).

**Conclusion:**

The use of corifollitropin alpha combined with a GnRH agonist for triggering is a safe, effective and acceptable protocol for oocyte donors.

## INTRODUCTION

Women’s reproductive fecundity is biologically age-limited and due to recent cultural shifts towards delayed childbearing, age-related infertility is the major reason underlying oocyte donation (Lutjen *et al.*, 1984). Apart from advanced maternal age, oocyte donation is a well-established mode of therapy for other infertility causes including: diminished ovarian reserve, repeated in vitro fertilization (IVF) failures, post-cancer infertility or maternally inherited genetic abnormalities (Sauer & Paulson, 1995).

In oocyte donation cycles, instead of the recipients, the oocyte donors undergo ovarian stimulation with gonadotropins to achieve multifollicular growth; therefore, issues of safety, treatment adherence and acceptability are of major importance. Taking into account that oocyte donors are selected from a young population with a high ovarian reserve, and although it is unusual to use high gonadotropin doses for stimulation, still there is an increased risk of ovarian hyperstimulation syndrome (OHSS) (Jayaprakasan *et al.*, 2007). This carries certain associated morbidity risks and sometimes leads to cycle cancellation, not to mention the increased risk of OHSS for the donor, which is both unpleasant and potentially dangerous (Hernández *et al.*, 2009). The GnRH antagonist protocol was a first evolution toward this goal. It is well documented that the risk of OHSS is almost two times lower when an antagonist is used compared with a long-agonist protocol (Al-Inany *et al.*, 2016).

Another innovation toward improving patient adherence with a more convenient IVF is the use of the long-acting FSH, corifollitropin alfa (Elonva^®^). Corifollitropin alfa is a recombinant glycoprotein with prolonged follicle-stimulating activity, where a single subcutaneous injection can initiate and sustain the growth of multiple follicles for the first 7 days of ovarian stimulation, reducing the number of injections required over the IVF cycle (Fauser *et al.*, 2011). This new FSH analogue has the same α-subunit as FSH, but its β-subunit has been extended by a carboxyterminal peptide of the hCG β-subunit. This results in the slower absorption and longer elimination half-life (65 hours) of corifollitropin alfa, contributing to its sustained duration of activity (Corifollitropin Alfa Dose-Finding Study Group, 2008). Moreover, corifollitropin alfa has the same pharmacodynamic effect as purified FSH as it only interacts with the FSH-receptor and lacks LH activity (Fauser *et al.*, 2011). However, only limited data exist regarding the use of corifollitropin alfa in donor cycles (Requena *et al.*, 2013).

The current study was designed to identify whether there are efficacy, safety and convenience advantages using long-acting FSH and GnRH agonist triggering in a population of oocyte donors.

## MATERIALS AND METHODS

### Population characteristics

This prospective longitudinal study including oocyte donation cycles was conducted between January 2014 to April 2019, at the Assisting Nature fertility center. The same eighty donors (n=80) underwent two consecutive antagonist stimulation schemes, involving 160 stimulation cycles; eighty cycles used the gold standard antagonist protocol of recombinant FSH (rFSH) plus hCG triggering and within 12 months, an additional eighty cycles with the new, more convenient protocol with long-acting FSH and GnRH agonist for triggering final oocyte maturation. The age of oocyte donors was between 23 and 33 years. They had a body mass index below 30 kg/m^2^, regular menstrual cycles, adequate ovarian reserve and no exclusionary medical history. A transvaginal scan was performed on all donors to exclude cases with polycystic ovaries, endometriosis or other pathological gynecological conditions. Hormonal evaluation including thyroid function, karyotype, testing for cystic fibrosis, full blood count, vaginal and cervical swabs. Screening for previous viral infections, including hepatitis B and C, human immunodeficiency virus, cytomegalovirus and syphilis was also done. All women included in the study provided informed consent for their participation. The Institutional Review Board reviewed the study protocol and approved the study procedure.

### Donor stimulation protocols

In donors following the first stimulation regimen, after a vaginal ultrasound examination and the confirmation of baseline FSH, LH and estradiol, stimulation commenced in the afternoon of Day 2 of the menstrual cycle with rFSH (200-300IU). The rFSH dose remained fixed until Day 6 of stimulation. Thereafter, the dose was adjusted according to the ovarian response. Daily GnRH antagonist co-treatment (Orgalutran^®^ 0.25 mg) was introduced from the morning of Day 6 of stimulation (Pacchiarotti *et al.*, 2016). Transvaginal ultrasound and blood sampling were performed on Day 6 of stimulation and thereafter as necessary until the day of triggering. Final oocyte maturation was induced with 250 µg recombinant hCG (r-HCG - Ovitrelle^®^), as soon as ≥3 follicles of ≥18 mm were present (Farrag *et al.*, 2008).

In the second stimulation scheme, one injection of a long-acting (7 days) FSH (Elonva^®^) was performed in the afternoon of Day 2 of the menstrual cycle. From Day 8 of stimulation until the day of triggering, FSH (Puregon^®^) was added according to ovarian response (Croxtall & McKeage, 2011). Daily GnRH antagonist co-treatment (Orgalutran^®^ 0.25 mg) was also administered from the morning of Day 6 of stimulation. After regular transvaginal ultrasound scans, final oocyte maturation was induced with the GnRH agonist Triptorelin (Arvecap^®^) 0.3 mg, as soon as ≥3 follicles of ≥18 mm were present (Croxtall & McKeage, 2011). Oocyte retrieval was carried out 36 h later, with aspiration of all follicles.

In both protocols, transvaginal ultrasound-guided oocyte pick-up was performed under intravenous sedation and local anesthesia, 36h after final oocyte maturation with r-hCG or GnRH agonist, followed by IVF or intracytoplasmic sperm injection (ICSI).

All donors answered a questionnaire with graded responses on a scale from 1 to 5 addressing four basic questions: (a) assessing their experience/discomfort during the follicular phase with each protocol, (b) their experience during the luteal phase, (c) whether they would repeat the treatment and (d) which protocol they would choose if they were allowed to do so. Each donor was asked to complete the questionnaire during the first menstruation after completing each scheme, thus two questionnaires were completed by each donor (Table 1).

**Table 1:** Questionnaire - rating of the donors after completing each protocol

*A. Please rate your adherence to the protocol (follicular phase):*
1. No adherence
2. Low adherence
3. Moderate adherence
4. Adequate adherence
5. High adherence
*B. Please rate your discomfort during the scheme (luteal phase):*
1. High discomfort
2. Tolerable discomfort
3. Moderate discomfort
4. Low discomfort
5. No discomfort
*C. How possible would you find it to repeat the treatment?*
1. Not possible
2. Not sure
3. Perhaps
4. Possible
5. Definitely
*D. Please rate the protocol in terms of convenience:*
1. Not convenient
2. Probably not convenient
3. Moderate convenience
4. Adequate convenience
5. Highly conveniente

**Rating** 1: no adherence, high discomfort, not possible, not convenient 2: low adherence, tolerable discomfort, not sure, probably not convenient 3: moderate adherence, moderate discomfort, perhaps, moderate convenience 4: adequate adherence, low discomfort, possible, adequately convenience 5: high adherence, no discomfort, definitely, highly convenient

### Recipient estrogen replacement protocols

In recipients who were amenorrheic after contraceptive pill discontinuation, bleeding was induced by receiving the estrogen replacement scheme as described below. In recipients who were still cycling, an estrogen replacement scheme was utilized starting during the 3^rd^ day of follicular phase (only a few downregulated since the 21^st^ day of the previous menstrual cycle using an intramuscular GnRH agonist). The estrogen replacement protocol was: on the 3^rd^ day of their period, the recipient was advised to start taking an estradiol regimen of estradiol valerate 2 mg with a gradually increasing dosing scheme (4 mg for 3 days, then 6 mg for 3 days and then 8 mg for the rest of the cycle) in order to mimic estradiol levels of the natural menstrual cycle. Estradiol was used until the pregnancy test, and if positive, until the 10^th^ week of pregnancy. Micronized progesterone was added after a minimum 10 days of estradiol pre-treatment and once the endometrium was thicker than 7 mm. In case of D3 embryo transfer, 200 mg progesterone (Utrogestan^®^) was administered to the recipient 4 days before embryo transfer. In case of D5 embryo transfer, 200 mg progesterone (Utrogestan^®^) was administered 6 days before blastocyst transfer. Progesterone was given intravaginally and was continued at 200 mg three times a day until the pregnancy test, and if positive, until the 10^th^ week of pregnancy. Methylprednisolone 8 mg and aspirin 100 mg were also co-administered in cases of proven thrombophilia or a background of autoimmune disease. Endometrial thickness was measured by ultrasound scan and it was considered mature above 7 mm. The pregnancy test was performed 14 days after initiation of progesterone.

### Outcomes

The primary outcomes were patient satisfaction (as scored by the questionnaire) and delivery rate per donor. Secondary outcomes were mean number of cumulus-oocyte-complexes, metaphase II (MII) oocytes and ovarian hyperstimulation syndrome (OHSS) rate.

### Statistical analysis

A sample size of 74 donors in each group was required to decrease the incidence of moderate and severe OHSS from 15% (already reported in the literature for IVF cases) with the classical antagonist with HCG triggering, to 1% with the proposed antagonist protocol with long-acting FSH and agonist triggering with an alpha value of 0.05 and a power of 90%.

Absolute (n) and relative (%) frequencies were calculated for categorical variables, while continuous variables were represented as mean ± standard deviation (SD). The statistical analysis for the comparison of the collected data was performed using McNemar’s test for categorical variables and Paired samples t-test for continuous variables. Statistical significance was defined as *p*<0.05. The analysis was performed using SPSS software v.25.0.

## RESULTS

In total, 80 oocyte donors performed 160 stimulation cycles, triggered in the initial cycle with r-hCG and in the second cycle with a GnRH agonist. In terms of donor experiences, donors rated the follicular phase adherence as 3.0 *versus* 1.1 (*p*<0.001) and the luteal phase discomfort as 3.1 *versus* 1.2 (*p*<0.001), in favor of the more convenient protocol (corifollitropin alpha+GnRH agonist triggering) compared with the conventional protocol (rFSH + hCG triggering). Moreover, donors rated themselves as being more inclined to repeat and to recommend the corifollitropin alpha + GnRH agonist triggering protocol than the conventional protocol (3.5 *versus* 1.5; *p*<0.001 and 3.4 *versus* 1.4; *p*<0.001, respectively) (1- high discomfort/not recommend to 5- low discomfort/strongly recommend) (Table 2).

**Table 2:** Questionnaire responses - rating of the donors after completing the two protocols

	rFSH +hCG trigger (n=80)	Corifollitropin alfa + GnRH agonist trigger (n=80)	Significance (Paired samples t-test)
Αdherence to the protocol (Follicular phase)	1.1	3.0	*p*<0.001
Luteal phase discomfort	1.2	3.1	*p*<0.001
Would you repeat the treatment	1.5	3.5	*p*<0.001
Which protocol would you recommend	1.4	3.4	*p*<0.001

(1- no adherence, high discomfort, not possible to repeat, not recommend to 5- high adherence, no discomfort, definitely repeat, strongly recommend)

Delivery rates were the same at 60.0% (48/80) in both groups. The clinical pregnancy rate per donor was comparable between the two groups, with 75.0% (60/80) after hCG-triggering, compared with 70.0% (56/80) after GnRH agonist triggering, *p*=0.13. Similarly, no significant differences were identified in the miscarriage rates (20.0% after hCG-triggering *versus* 14.3% after GnRH agonist triggering; *p*=0.14) (Table 3).

**Table 3:** Comparison of oocyte donor cycles using rFSH + hCG or corifollitropin alfa + GnRH-agonist trigger

	rFSH + hCG trigger (n=80)	corifollitropin alfa + GnRH agonist trigger (n=80)	Significance
Age (years)	24.3	25.1	NS (Paired samples t-test)
Delivery rate per donor	60.0%(n=48/80)	60.0%(n=48/80)	NS (McNemar’s test)
Clinical pregnancy rate per donor	75.0%(n=60/80)	70.0%(n=56/80)	NS (McNemar’s test)
Miscarriage rate	20.0%(n=12/60)	14.3%(n=8/56)	NS (McNemar’s test)
COCs (mean± SD)	17.6±9.5	16.3±7.8	NS (Paired samples t-test)
MII oocytes (mean±SD)	13.1±8.2	12.3±6.7	NS (Paired samples t-test)
Number of transferred embryos (mean± SD)	2.0±0.3	2.0±0.5	NS (Paired samples t-test)
OHSS (n %)	3.75%(n=3)	0%(n=0)	NS (McNemar’s test)
Luteal phase duration (days)	10.2	5.1	*p*<0.001 (Paired samples t-test)

NS: not significant, OHSS: ovarian hyperstimulation syndrome, SD: standard deviation, COCs: cumulus-oocyte-complexes, MII: metaphase II

Regarding the secondary outcomes, no difference was identified between the two protocols in the number of oocytes (17.6±9.5 with rFSH+hCG triggering *versus* 16.3±7.8 with corifollitropin alfa + GnRH agonist triggering, *p*=0.35), the number of MII oocytes (13.1±8.2 with rFSH+hCG triggering *versu*s 12.3±6.7 with corifollitropin alfa + GnRH agonist triggering, p=0.50) and the number of transferred embryos (2.0±0.3 with rFSH + hCG *versus* 2.0±0.5 with corifollitropin alfa + GnRH agonist). Three cases of moderate OHSS (3.75%) were identified after hCG triggering, but no case of OHSS occurred after GnRH agonist triggering (*p*=0.25). None of the donors developed severe OHSS requiring hospitalization. Nevertheless, the duration of the luteal phase was significantly shorter after GnRH agonist (5.1 days) compared to hCG triggering (10.2 days), *p*<0.001 (Table 3).

## DISCUSSION

This prospective longitudinal study was carried out in 80 donors who underwent two consecutive antagonist stimulation schemes; one using the classical antagonist protocol with rFSH plus hCG for triggering and a subsequent one with an innovative antagonist protocol combining the use of corifollitropin alfa with a GnRH agonist for triggering. The latter protocol proved equally effective in terms of oocyte yield, transferrable blastocysts produced and eventually pregnancy outcomes in oocyte acceptors, and, in addition to the excellent acceptability for donors, achieved a high degree of adherence with a very low degree of discomfort. All these aspects suggest that corifollitropin alfa and GnRH agonist for triggering is a more acceptable IVF protocol for donors, combining safety, efficacy and simplicity.

Regarding corifollitropin alfa, a single injection of this long-acting FSH on the first day of stimulation can replace the first seven daily injections of rFSH, simplifying treatment and making assisted reproduction more acceptable of patients; which for donors can be of particular importance, especially during a first treatment when they may be nervous or afraid as they have had no previous experience of the procedure. In fact, when donors were asked to choose which treatment they preferred, the results clearly showed a positive trend favoring corifollitropin alfa, suggesting that this new protocol may reduce the treatment burden and increase donor adherence. This finding is in accordance with evidence from other studies, confirming donors’ preferences (Requena *et al.*, 2013).

In terms of the efficacy of corifollitropin alfa, in the present study the replacement of daily rFSH with a single injection of corifollitropin alpha in the subsequent cycle had no impact either on embryological or pregnancy outcomes, as the new acceptors had identical pregnancy rates as the first acceptors who had taken blastocysts produced with 9-12 daily injections of rFSH. Similarly, three randomized control trials of women undergoing ovarian stimulation with either corifollitropin alfa or rFSH showed that the use of a single injection of corifollitropin alfa for the first seven days of ovarian stimulation was either equivalent or non-inferior to daily injections of rFSH in terms of the number of oocytes retrieved, and in terms of pregnancy and live-birth rates. Moreover, there were no significant differences in the incidences of OHSS between corifollitropin alfa and rFSH in these three trials (Devroey *et al.*, 2009; Corifollitropin Alfa Ensure Study Group, 2010; Boostanfar *et al.*, 2015).

Regarding pregnancy outcomes, the first meta-analysis in a normal IVF population showed a lower likelihood of achieving a clinical pregnancy with GnRH agonist triggering in a GnRH antagonist protocol with standard luteal phase support with estrogen and progesterone (Griesinger *et al.*, 2006). Conversely, despite the fact that a meta-analysis by Humaidan *et al*. (2011) showed no difference in the delivery rates if intense luteal support is administered, the fresh embryo transfer policy after agonist triggering is not widely used for the normal IVF population as the freeze-all strategy has subsequently emerged and has eliminated the need for intense luteal support.

However, these cautions do not affect the current study’s population, namely of oocyte donors. In fact, in the present study, the delivery rates were equal in both groups.

In order to exclude the possible negative impact of the corpus luteum and endometrium in a GnRH triggered cycle, Acevedo *et al*. (2006) examined this mode of oocyte maturation in a donor programme evaluating the number of retrieved oocytes, MII oocytes, fertilization, pregnancy and implantation rates. The results were equivalent compared with hCG triggered donor cycles, suggesting that embryo quality is not affected by GnRH agonist triggering (Acevedo *et al.*, 2006). Moreover, similarly to the results of the present study, significant differences in luteal phase length (4.16±0.70 days *versus* 13.63±2.12 days) and in OHSS (0/30 *versus* 5/30) were observed between donors receiving a GnRH agonist compared with those receiving hCG (Acevedo *et al.*, 2006).

Importantly, the emergence of OHSS was eliminated with the GnRH agonist triggering scheme compared with the use of hCG-triggering. Naturally this is a significant benefit in an IVF cycle, especially in young, healthy, altruistic oocyte donors. The need for a low-risk OHSS protocol necessitated the implementation of GnRH antagonist protocols, which subsequently paved the way for the introduction of a GnRH agonist for triggering oocyte maturation (Humaidan *et al.*, 2011). The GnRH agonist as a trigger module has a shorter half-life and elicits a more physiological flare-up of gonadotropins, thus it appears to be an appropriate first-line regimen for final oocyte maturation in donor stimulation cycles (Youssef *et al.*, 2015). In the initial studies, a GnRH agonist was also proposed as an alternative triggering agent for women at increased risk of OHSS, such as oocyte donors (Shapiro *et al.*, 2007). In the study of Bodri *et al*. (2009), in 2,077 donor cycles the triggering agent was selected according to the follicular number on the day of triggering and the pregnancy outcomes were not statistically significantly different, with data also supporting the use of GnRH in order to reduce the risk of OHSS. In another randomized trial including 212 oocyte donors, half of whom received oocyte triggering with hCG and half with GnRH agonist, fertilization rates were similar but the incidence of OHSS in the second group was considerably reduced (Galindo *et al.*, 2009).

As mentioned previously, the duration of the luteal phase was significantly shorter after GnRH agonist triggering compared with hCG triggering, thus donors following this mode of oocyte maturation triggering, can more easily re-establish their regular menstrual cycle and experience less pelvic discomfort, increasing the likelihood of participation in future donation cycles. This shorter duration of the luteal phase is also indirect evidence of impaired luteal function induced by GnRH agonist triggering (Humaidan *et al.*, 2012; Fatemi *et al.*, 2013); however this has no negative effects on oocyte-acceptor cycles when the recipient’s endometrium is appropriately prepared.

The major strength of this study was that two consecutive stimulation schemes were evaluated in the same donors, minimizing potential selection bias. Additionally, to the best of our knowledge, this is the first study using a long-acting FSH combined universally in an oocyte donor population compared head-to-head with the classical antagonist and rFSH plus hCG triggering protocol.

However, the study was limited to a population of European donors. In addition, the study was conducted at a single center, so the results may not be generalizable to a wider population. Moreover, the use of methylprednisolone or aspirin could work as a confounder. However, only one patient received methylprednisolone and two patients received aspirin due to proven antiphospholipid syndrome; this effect may be insignificant as the pregnancy rates were equivalent between study groups. Finally, using oocyte donors as study participants may result in a less diverse study population, making baseline characteristics less prominent than they would be in an infertile population undergoing IVF.

The antagonist protocol with long-acting FSH and agonist triggering appeared to meet the requirements for safety, efficacy and simplicity; each of which is essential for the oocyte-donor population. In donor cycles, the absence of pregnancy in donors may exclude the possibility of late OHSS; however, as the enrolled women were younger and with good reproductive potential, there is an increased risk of early OHSS. The present study emphasises the need in oocyte donors to use - as the safest reproductive treatment - the combination of antagonist downregulation, long-acting FSH for follicular stimulation and agonist triggering. This strategy has now been shown to not only achieve a desirable oocyte yield, but also to minimize the risk of OHSS and to reduce the recovery time from an IVF-stimulated cycle.

## CONCLUSION

In order to help make the dream of parenthood come true for oocyte-acceptors, it is essential that the treatment that oocyte donors undergo is as safe, effective and convenient as possible. The antagonist protocol with long-acting FSH and agonist triggering is both more acceptable and safer for oocyte donors and appears to be an appropriate approach for first-line treatment in oocyte-donation programs.
